# Shifty salamanders: transient trophic polymorphism and cannibalism within natural populations of larval ambystomatid salamanders

**DOI:** 10.1186/s12983-014-0076-7

**Published:** 2014-10-14

**Authors:** Dale M Jefferson, Maud CO Ferrari, Alicia Mathis, Keith A Hobson, Eric R Britzke, Adam L Crane, Andrew R Blaustein, Douglas P Chivers

**Affiliations:** Department of Biology, University of Saskatchewan, 112 Science Place, S7N 5E2 Saskatoon, SK Canada; Department of Biomedical Sciences WCVM, University of Saskatchewan, 52 Campus Dr., S7N 5B4 Saskatoon, SK Canada; Department of Biology, Missouri State University, 65897-0095 Springfield, MO USA; Environment Canada, S7N 3H5 Saskatoon, SK Canada; Department of Zoology, Oregon State University, 97331 Corvallis, OR USA

**Keywords:** Cannibalism, Larval salamander, Carbon-13, Trophic polymorphism, Nitrogen-15, Stable isotope

## Abstract

**Introduction:**

Many species of ambystomatid salamanders are dependent upon highly variable temporary wetlands for larval development. High larval densities may prompt the expression of a distinct head morphology that may facilitate cannibalism. However, few studies have characterized structural cannibalism within natural populations of larval salamanders. In this study we used two species of larval salamanders, long-toed (*Ambystoma macrodactylum*) and ringed salamanders (*A. annulatum*). Head morphometrics and stable isotopic values of carbon (δ^13^C) and nitrogen (δ^15^N) were used to identify the presence or absence of structural cannibalism. Weather conditions were also analyzed as a potential factor associated with the expression of cannibalistic morphology.

**Results:**

Populations of salamander larvae did not consistently exhibit cannibalistic morphologies throughout collection periods. Larval long-toed salamanders exhibited trophic polymorphisms when relatively lower precipitation amounts were observed. Larval ringed salamanders were observed to be cannibalistic but did not exhibit polymorphisms in this study.

**Conclusions:**

Structural cannibalism may be transient in both species; however in long-toed salamanders this morphology is necessary for cannibalism. Ringed salamanders can be cannibalistic without morphological adaptations; however the cannibal morph may prolong the viable time period for cannibalism. Additionally, weather conditions may alter pond hydroperiod, subsequently influencing head morphology and cannibalism.

## Introduction

Temporary wetlands are important habitats to the larval development of many species of amphibians [1]. However, such habitats inevitably undergo pond drying imposing temporal and/or spatial limitations upon developing larvae [1,2]. Wetland drying increases the risk of desiccation while simultaneously increasing larval density and potentially limiting food resource availability [2]. These restrictions can force larvae into feeding aggregations, increasing the degree of intraspecific competition, and leading to aggression and intraspecific predation [2–5].

Larval amphibians exhibit sensitivity to intraspecific competition and may express specific morphological and/or behavioural adaptations leading to improved foraging success and increased rate of development, which allows for larvae to survive and escape inclement conditions [3,6–11]. Many species of larval amphibians may exhibit morphological adaptations that could improve foraging success or alter the prey they consume [e.g., 12–15]. In larval ambystomatid salamanders such adaptations include enlarged feeding structures (i.e. jaws and teeth), which increase the prey size larvae are capable of consuming, and in turn increases their potential trophic niche width [16,17]. These structural polymorphisms have also been associated with cannibalistic behaviour because they facilitate the consumption of similarly sized conspecifics [18,19].

Here, we used two model species of larval salamanders (*Ambystoma macrodactylum* and *A. annulatum*) from populations previously observed to exhibit the “cannibalistic morphology” [18,19]. Both species are explosive breeders as they both produce large numbers of offspring over a short period of time and both utilize temporary wetlands for breeding and larval development [20]. However, these species differ in key aspects of their breeding strategy. Long-toed salamanders tend to breed synchronously, while ringed salamanders often breed over a period of a month or more [20]. This difference results in an age and size hierarchy within larval populations of ringed salamanders while populations of larval long-toed salamanders tend to be of similar age and are at least initially of similar size [20].

Studies documenting trophic polymorphism have primarily shown cannibalistic behaviour under laboratory conditions [19]. However, Nyman et al. [19] characterized the difference in head morphology of larval ringed salamanders between cannibals and non-cannibals based on gut content analysis from individuals in a natural population. The purpose of our study was to further test the linkage between cannibalism and morphology in two species that produce larvae exhibiting the “cannibalistic morph”. Additionally, since neither larval densities nor pond conditions are static in natural populations we expect that expression of polymorphisms and/or cannibalism may be transient within these populations among years [18]. Larval long-toed salamanders from Oregon were tested using morphological, and carbon (δ^13^C) and nitrogen (δ^15^N) stable isotopic data to compare differences in morphology with differences in trophic niche occupation. Larval ringed salamanders from Missouri were differentiated into cannibals and non-cannibals based on gut-content analysis and compared for differences in head morphology and δ^13^C and δ^15^N values. Climate data for collection sites were also used to identify weather patterns that may have influenced pond condition and subsequently the ecology of salamander larvae.

## Results

### Long-toed salamanders

A total of 98 larvae (June 2007 n = 24; August 2007 n = 14; July 2008 n = 21; August 2008 n = 39) were sub-sampled from the 197 larvae collected between 2007 and 2008. Results of the PCA of the two sampling years indicated that the first two components described 98.5% of the total variation among variables (Table 1). The first principal component (PC1) described the vast majority of the total variance (97.3%) and described variance in overall size. The second principal component (PC2) described 1.2% of the total variance and characterized head shape. This component described variation in PREHW. The highly negative value of the eigenvector loading indicates that increasing values of PC2 corresponds to a decreasing PREHW. No significant difference was observed in the overall sizes between salamander larvae collected in 2007 and those collected in 2008 as characterized through a *t*-test of PC1; however a subsequent *t*-test identified a significant difference in PC2 (*t*_[89]_ = -5.6; *P* <0.001).

**Table 1:** **Eigenvectors for each morphometric in each principle component and percent of total variation explained by each principle component**

	**Eigenvectors**	
Variable	PC1	PC2
Mass	**0.449**	0.257
HL	**0.449**	0.313
MHW	**0.448**	0.465
SVL	**0.446**	-0.328
PREHW	**0.444**	**-0.716**
Percent of total variation	97.4	1.2

Bold values denote important variable contributions to principle components.

**Figure 1:**
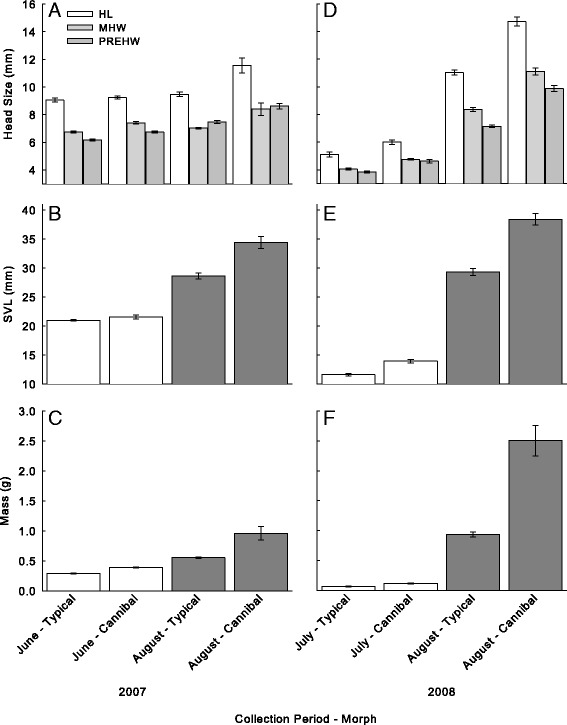
**Mean (±SE) morphometrics of putative typical and cannibal morph larval long-toed salamanders (grouped based on head morphometrics) collected in 2007: head length, maximum head width, and pre-ocular head width (A), snout-vent length (B), and mass (C); and 2008: head length, maximum head width, and pre-ocular head width (D), snout-vent length (E), and mass (F).**

**Figure 2:**
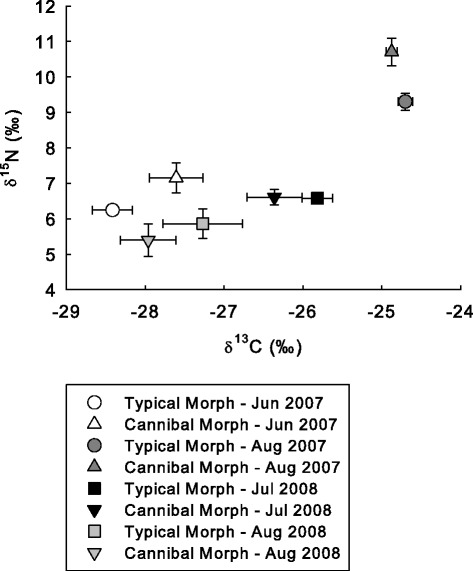
**Mean (±SE) δ** ^**13**^ **C and δ** ^**15**^ **N values of putative typical and cannibal morph (grouped based on head morphometrics) larval long-toed salamanders.**

Multivariate analysis of salamanders collected in June 2007 clustered based on head morphometrics (Figure 1A) exhibited significant overall differences in SVL, mass, δ^13^C and δ^15^N (Hotelling-Lawley trace: *F*_[4,19]_ = 12.2; *P* <0.001). Univariate *F*-tests indicated that larvae classified in the cannibal group exhibited significantly larger SVL (*F*_[1,22]_ = 7.2; *P* = 0.01), mass (*F*_[1,22]_ = 56.1; *P* <0.001; Figure 1B-C), and δ^15^N (*F*_[1,22]_ = 5.9; *P* <0.05; values summarized in Table 2). However, no significant difference was observed in δ^13^C values between cannibal and typical groups of salamanders (Table 2; Figure 2). When these salamanders were re-clustered based on δ^15^N values, multivariate analysis identified a significant difference between the putative cannibal and typical groups (Wilk’s Lambda = 0.5; *F*_[3,13]_ = 7.3; *P* <0.05). Subsequently, discriminant analysis of the transformed head morphometrics correctly classified 100% of typical morphs (13 of 13) and 50% of cannibal morphs (2 of 4) with a total correct classification of 88%.

**Table 2:** **Mean (±SE) values of mass, snout-vent length (SVL), δ** ^**13**^ **C, and δ** ^**15**^ **N for larval long-toed salamanders clustered into putative cannibal or typical morphology groups based on head morphometrics collected in June and August 2007, and July and August 2008**

**Year**	**Month**	**Morphology**	**n**	**Mass (g)**	**SVL (mm)**	**δ** ^**13**^ **C (‰)**	**δ** ^**15**^ **N (‰)**
2007	June	Cannibal	4	0.4 ± 0.0	21.5 ± 0.3	-27.6 ± 0.3	7.2 ± 0.4
		Typical	12	0.3 ± 0.0‡	20.9 ± 0.1‡	-28.4 ± 0.2	6.2 ± 0.1*
	August	Cannibal	3	0.9 ± 0.1	34.4 ± 1.1	-24.7 ± 0.1	10.7 ± 0.4
		Typical	11	0.6 ± 0.0†	28.6 ± 0.6†	-24.9 ± 0.1	9.3 ± 0.2*
2008	July	Cannibal	12	0.1 ± 0.0	13.9 ± 0.3	-27.9 ± 0.4	5.4 ± 0.4
		Typical	9	0.1 ± 0.0†	11.6 ± 0.2†	-27.3 ± 0.5	5.8 ± 0.4
	August	Cannibal	14	2.5 ± 0.3	38.4 ± 1.0	-26.4 ± 0.4	6.6 ± 0.2
		Typical	29	0.9 ± 0.0†	29.2 ± 0.6†	-25.8 ± 0.2	6.6 ± 0.1

Symbols denote significant differences between putative cannibal and typical groups for each sampling period: † ≤0.001; ‡ ≤0.01; * ≤0.05.

Multivariate analysis of salamanders collected in August 2007 identified overall differences between individuals grouped based on head morphometrics where predicted PREHW values were included (Figure 1A) (Hotelling-Lawley trace: *F*_[4,9]_ = 12.2; *P* = 0.001). Univariate F-tests subsequently identified significant differences in mean SVL (*F*_[1,12]_ = 20.2; *P* = 0.001), mass (*F*_[1,12]_ = 46.6; *P* <0.001; Figure 1B-C), and δ^15^N values (*F*_[1,12]_ = 46.6; *P* <0.05; values summarized in Table 2). No significant difference was observed in mean (±SE) values of δ^13^C between groups (Table 2; Figure 2). When re-classified based on δ^15^N values, multivariate analysis identified significant differences between groups in transformed head morphometrics (Wilk’s Lambda = 0.4; *F*_[3,10]_ = 4.7; *P* <0.05). Discriminate analysis correctly classified a total of 93% of salamanders with 100% of the typical morphs being correctly identified (7 of 7) and 86% of cannibal morphs were correctly classified (6 of 7). Where predicted values of PREHW were excluded from analyses, an overall difference between groups (classified based on head morphology, excluding PREHW) was still identified using multivariate analysis (Hotelly-Lawley: *F*_[4,9]_ = 14.2; *P* = 0.001). Univariate *F*-tests also observed significant differences in SVL (*F*_[1,12]_ = 15.7; *P* <0.005) and mass (*F*_[1,12]_ = 47.6; *P* <0.001); however no significant differences were observed between groups in δ^13^C and δ^15^N values. No significant differences were observed between groups (classified based on δ^15^N values) using multivariate analysis of transformed head morphometrics where PREHW values were excluded.

Significant differences were observed between salamanders grouped based on head morphometrics using multivariate analyses in both July (Hotelling-Lawley trace: *F*_[4,16]_ = 8.0; *P* = 0.001) and August (Figure 1D) (Hotelling-Lawley trace: *F*_[4,34]_ = 29.1; *P* <0.001) 2008. Univariate *F*-tests identified significant differences between cannibal and typical morph groups in SVL (July: *F*_[1,19]_ = 34.4; *P* <0.001; August: *F*_[1,37]_ = 66.4; *P* <0.001; Figure 1E) and mass (July: *F*_[1,19]_ = 24.2; *P* <0.001; August: *F*_[1,37]_ = 121.1; *P* <0.001; values summarized in Table 2; Figure 1F). However, no significant differences were observed in δ^13^C or δ^15^N (values summarized in Table 2; Figure 2) in either month. When salamander larvae were re-classified into groups based on δ^15^N values no significant differences were observed through multivariate analyses between cannibal and typical groups in either July or August. Multivariate analyses indicated no significant differences between salamander groups in overall head morphometrics in July or August.

**Figure 3:**
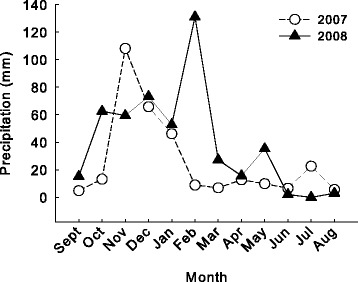
**Monthly mean (±SE) precipitation data for Sisters, OR between September 2006 – August 2007, and September 2007 – August 2008.**

Multivariate comparison of weather data identified no overall significant difference between years. However, while not statistically different the mean temperature was relatively lower in 2008 relative to that of 2007). Conversely, the mean (±SE) precipitation was lower in 2007 than in 2008 and the sum of precipitation was more than 150 mm lower in 2007 relative to the same time period in 2008 (values summarized in Table 3). Additionally, there was a greater influx of precipitation in early spring in 2008, which may have maintained or increased pond size during early larval salamander development (Figure 3).

**Table 3:** **Mean (±SE) temperature and precipitation, and total precipitation data for Sisters, OR between September 2006 – August 2007 and September 2007 – August 2008, and for Reeds Spring, MO between October 1983 – May 1984, and October 1994 – May 1995**

**Location**	**Year**	**Mean temperature (°C)**	**Mean precipitation (mm)**	**Total precipitation (mm)**
Oregon	2007	8.1 ± 2.1	25.9 ± 9.2	311.4
	2008	7.2 ± 2.1	39.8 ± 11.1	477.4
Missouri	1983	7.6 ± 2.7	104.1 ± 15.8	832.6
	1994	9.2 ± 1.9	115.9 ± 26.6	927.4

### Ringed salamanders

Of the 669 salamander larvae collected 124 larvae were sub-sampled for stable isotope and statistical analysis. A total of 14 salamander larvae were identified as definitively cannibals based on gut contents from the total collection and were only observed between collection weeks 3-16 (November 2, 1994 – February 7, 1995). A total of 73 non-cannibals collected from the same time period as the observed cannibals, were randomly selected from the total collection. A total of 37 hatchling salamanders were collected from weeks 1 and 2 (October 20 – 28, 1994) and were used as approximations for putative conspecific prey in analyses of morphometrics and stable isotope values.

**Figure 4:**
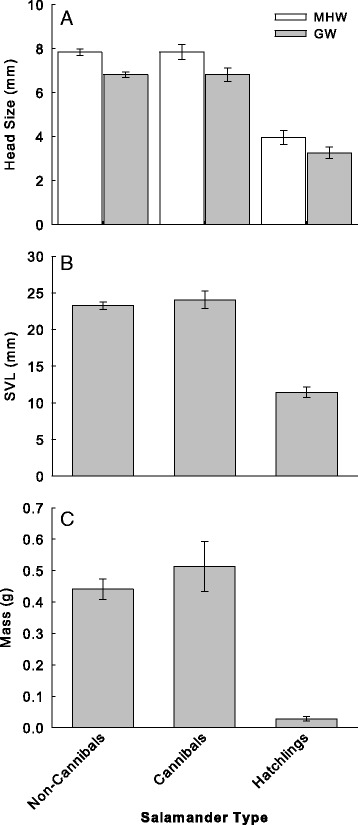
**Mean (±SE) morphometrics of cannibal, non-cannibal, and hatchling larval ringed salamanders.** Maximum head width, and gape width **(A)**, snout-vent length **(B)**, and mass **(C)** collected between October 1994 – February 1995.

Among the three groups of salamanders overall significant differences were observed in SVL (*F*_[2,123]_ = 209.2; *P* <0.001), mass (*F*_[2,123]_ = 137.4; *P* <0.001), MHW (*F*_[2, 123]_ = 165.4; *P* <0.001), and GW (*F*_[2, 123]_ = 167.9; *P* <0.001). Pairwise comparisons identified no difference between cannibals and non-cannibals in SVL, mass, MHW or GW (all *P* >0.05; values summarized in Table 4). Hatchlings were significantly smaller in SVL, mass, MHW, and GW relative to both cannibals and non-cannibals (all *P* <0.001; summarized in Table 4; Figure 4). These results further validate that hatchlings represent potential prey as their mean MHW (the widest portion of their body) of hatchlings is smaller than the mean gape width of both cannibals and non-cannibals (Figure 4A).

**Table 4:** **Mean (±SE) values of mass, snout-vent length (SVL), maximum head width (MHW), gape width (GW), δ** ^**13**^ **C, and δ** ^**15**^ **N among cannibalistic, non-cannibalistic, and hatchling larval ringed salamanders**

**Group**	**n**	**Mass (g)**	**SVL (mm)**	**MHW (mm)**	**GW (mm)**	**δ** ^**13**^ **C (‰)**	**δ** ^**15**^ **N (‰)**
Cannibal	15	0.5 ± 0.1^a^	24.1 ± 1.2^a^	7.8 ± 0.3^a^	6.8 ± 0.3^a^	-21.7 ± 0.4^a^	7.5 ± 0.3^a^
Non-cannibal	73	0.4 ± 0.0^a^	23.3 ± 0.5^a^	7.8 ± 0.1^a^	6.8 ± 0.1^a^	-22.6 ± 0.3^a^	7.4 ± 0.1^a^
Hatchling	38	0.03 ± 0.01^b^	11.4 ± 0.7^b^	3.5 ± 0.2^b^	2.9 ± 0.2^b^	-21.9 ± 0.3^b^	5.4 ± 0.2^b^

Different letters denotes significant difference between groups.

**Figure 5:**
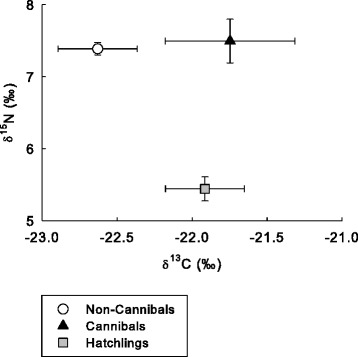
**Mean (±SE) δ** ^**13**^ **C and δ** ^**15**^ **N values among cannibal, non-cannibal, and hatchling ringed salamander larvae.**

Multivariate analysis of stable isotopic values of the three salamander groups identified an overall significant difference among these groups (Hotelling-Lawley trace: *F*_[4, 242]_ = 33.1; *P* <0.001). Univariate *F*-tests identified no significant differences among groups in δ^13^C values; however an overall significant difference was observed among groups in δ^15^N values (*F*_[2, 123]_ = 64.7; *P* <0.001; Table 4). Pairwise comparisons identified significant differences in δ^15^N values between hatchlings with both cannibals and non-cannibals (both *P* <0.001; Table 4; Figure 5); however no significant difference was observed between cannibals and non-cannibals.

Comparison of head morphology between cannibal and non-cannibal larvae identified no difference in the ratios of MHW or GW to SVL (equivalent slopes). No differences in either head morphometric were observed when adjusted for differences in SVL.

**Figure 6:**
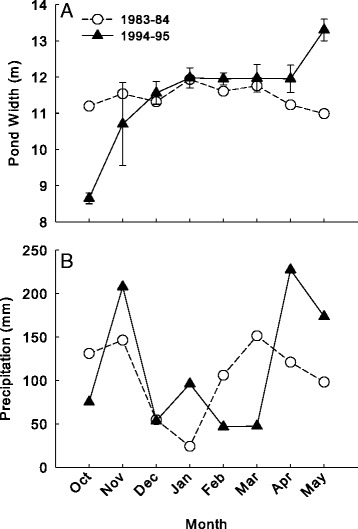
**Pond widths of Kirby’s pond, Reeds Spring, MO and monthly mean precipitation in the area.** Observed pond widths from October 1994 – May 1995 and the predicted pattern of pond width over the same time period in 1983-84 **(A)**, and the monthly mean precipitation observed in the area for both time periods **(B)**.

No significant difference in overall weather was observed between years through multivariate analysis. However, mean (±SE) precipitation and temperature was higher in 1994-95 relative to 1983-84 . Similarly, the total observed precipitation was nearly 100 mm greater in 1994-95 relative to 1983-84 (results summarized in Table 3). Predictive regression suggested that pond width potentially remained relatively consistent or shrank slightly from October 1983 to May 1984 based on weather data from this period. Nyman et al. [19] observed that the pond had a maximum surface area of 150 m^2^ and a depth of ≤70 cm and this was reduced through the summer. Conversely, pond measurements recorded over the 1994-1995 collection period show an increase in pond width by approximately 5 m from October 1994 (mean ± SE: 8.6 ± 1.5 m) to May 1995 (mean ± SE: 13.3 ± 0.3 m; Figure 6A). Additionally, we observed relatively high precipitation in November 1994, which corresponds with the initial increase in pond size. Similarly, the additional influx of precipitation during the colder winter months and early spring appear to maintain and increase pond size throughout the developmental period of these larvae (Figure 6A,B).

## Discussion

The results of this study suggest that a linkage between cannibalism and head morphology occurs within natural populations of both species of larval salamanders. However, the necessity for enlarged head morphology to facilitate cannibalism appears related to the breeding strategies of salamanders. Additionally, our results suggest the expression of polymorphisms within these larval populations is transient and potentially related to the hydrological condition of natal ponds.

Food availability and conspecific density influence the expression of intraspecific aggression and cannibalistic behaviour [2,4,5,21,22]. Where larval salamander densities are naturally high, and/or where pond conditions act to increase larval densities (i.e. pond drying) larvae may be forced into aggregations around limited food resources leading to increasingly high degrees of competition [2,23]. Peacor and Pfister [8] indicated that intra-population size variation of larval amphibians raised at high population densities resulted from phenotypic adaptations (‘size-independent’ factors) causing differences in foraging efficiency among individuals [24]. Acquisition of limited resources can therefore be improved in individuals with these advantageous traits [8]. The effects of these traits then become increasingly pronounced with increasing competition, and thus an increased size disparity develops among individuals within the population [8]. Therefore, increasing density could potentially result in the expression of a specialized head morphology that facilitates cannibalism. Simultaneously high larval density could provide the opportunity for cannibalism by forcing putative cannibals and their prey into proximity of each other.

Our results suggest larval long-toed salamanders exhibited variation in their head morphologies in 2007 (Figure 1A), and those individuals exhibiting the larger head morphology occupied a significantly higher trophic position based on δ^15^N values. Additionally, the observation that putative cannibals exhibited a significantly greater mass than typical larvae was similarly observed by Wildy et al. [25]. These results suggest the presence of a linkage between cannibalism and head morphology. However, no such relationship was observed among larval salamanders collected from the same wetland in 2008. This difference may be due to the concurrent breeding strategy of long-toed salamanders [20]. Since these salamanders breed explosively, all larvae within a population should be approximately the same age and of similar size. Petranka and Thomas [26] suggest that the evolution of synchronous breeding was influenced by the efficiency of larval amphibian cannibalization of vulnerable conspecifics. Indeed, synchronized breeding can reduce the risk of cannibalism by limiting the differences in size and development among individuals [26,27]. Therefore, specific adaptations in head morphology (i.e. the cannibal morphology) may greatly facilitate the ability of individuals of this species to consume conspecifics [12,14,28,29]. The greater gape size of the putative cannibal morphs provides these individuals an initial benefit by improving their ability to consume larger prey, including similarly sized conspecifics [28]. Successful cannibals may experience subsequent predation success resulting from the increased growth facilitated by cannibalism [25,29,30].

Our observations of larval ringed salamanders appear to contradict those of Nyman et al. [19] who found significant differences in head morphology and shape between cannibals and non-cannibals. Additionally, Nyman et al. [19] observed the presence of cannibalistic larvae within the pond until April, approximately to the observed initiation of metamorphosis. Conversely our last observation of cannibalism occurred in February. Ringed salamanders breed over a period of a month or more, providing a natural size differentiation among larvae of different age classes [20]. This moderately extended breeding period may allow cannibalism to occur without the expression of specific head morphologies [19,20]. However, it is possible the expression of larger head morphology could prolong the period during which larvae can consume conspecifics. Alternatively, if larval densities were sufficiently low the occurrence of cannibalism may also be reduced due to insufficient opportunity [23]. Similarity in stable isotopic values between cannibal and non-cannibal groups could be the result of one or more of the following situations: 1) gut content analysis provides only a snap-shot of feeding; cannibalism may have occurred but was not observed in members of the non-cannibal group because conspecific prey were completely digested prior to capture; 2) cannibalistic individuals were observed to have ingested conspecifics but had not digested this prey and therefore would not have assimilated this diet into their tissues; and 3) cannibalism does not represent a significant contribution to the overall diet of salamanders and therefore does not significantly alter the isotopic values of cannibals [31]. The most probable cause of this result is that cannibalism represents a relatively low proportion of the larval salamander diet. Conspecific prey was sufficiently large to fill and/or exceed the upper digestive tract of the observed cannibals. This suggests that digestion would take much longer than smaller invertebrates, which make up the majority of larval salamander diet [19]. Therefore, it is possible that cannibalism is relatively common among individuals; however the frequency of cannibalistic behaviour in any individual would be relatively low.

The transient nature of these trophic polymorphisms may have been, in part, related to the differences in precipitation between collection years. Brooks [32] observed that the water level of temporary wetlands was significantly related to precipitation. We observed that while there was no significant difference in precipitation or temperature between collection years in either study, the sum of precipitation was greater in years where cannibalistic morphs were absent. More importantly, the timing of the observed influxes of precipitation suggests that pond size may have been maintained or increased in cases where no differentiation in head morphology was observed among larval salamanders. Our results appear consistent with those that may be expected. However, the absence of comprehensive data regarding larval densities, prey abundance, and pond conditions during all sampling periods means it is not possible to definitively conclude this timing as evidence of a causative relationship.

The results of this study support the linkage between morphology and cannibalism in larval salamanders within natural populations and the density dependent nature of this relationship. The expression of rapid phenotypic adaptations to facilitate cannibalism may be the result of increasing competition [3,5–7,18]. Differences in life history among salamander species and weather patterns may also have important consequences for the expression of polymorphisms and cannibalism. The ubiquitous assignment of polymorphism across larval salamander species that exhibit cannibalism may therefore be inaccurate.

If these assumptions are correct there may be serious implications to population dynamics and survivorship of larvae that could result from changes in climate and/or habitat [32]. Long-term monitoring of polymorphic larvae in natural populations, and comparison across multiple species may therefore be necessary to develop a more complete understanding of the dynamics of this phenomenon.

## Conclusions

Trophic polymorphisms are a potential competitive adaptation expressed by larval salamanders under conditions of high competition. Concurrent breeding species, such as long-toed salamanders may be dependent upon such polymorphisms to facilitate intraspecific predation. Species that are not concurrent breeders may be cannibalistic without exhibiting the polymorphisms due to size differentiation inherent within the population; however individuals exhibiting the cannibal morphology may be capable of intraspecific predation for longer periods of time. Additionally, it appears as though the expression of these trophic polymorphisms may be influenced by seasonal precipitation and temperatures. Therefore, trends towards warmer drier climates could influence population dynamics of larval salamanders resulting in increased expression of trophic polymorphisms and cannibalistic behaviour.

## Materials and methods

### Field collections

#### Long-toed salamanders

Long-toed salamander larvae (*A. macrodactylum*) were collected with net sweeps performed from along the pond shoreline at two sampling periods in June and August 2007, and in July and August 2008 from an ephemeral montane pond located at an altitude of 1951 m above sea level in the central Cascade Mountains, 24.2 km south of Sisters, Deschutes County, Oregon. All larvae were physically euthanized by pithing, measured for head and body morphometrics with vernier calipers (to 0.1 mm), and frozen. Salamander larvae were measured for head length (HL; tip of snout to attachment point of first pair of gills), maximum head-width (MHW; width across the head at its widest point), snout-vent length (SVL; length from tip of the snout to the anterior end of the vent), and pre-ocular head width (PREHW; width across the head through bisecting line through the external nares). Data for PREHW of salamanders collected in August 2007 was lost and was therefore estimated from regression analysis of these characteristics against SVL from all other long-toed salamander used in this study. Gut content analysis of these specimens was not possible due to the physical degradation that occurred from frozen storage. Specimens were delivered to the University of Saskatchewan in August, 2010.

Climate data of the collection area (near Sisters, Oregon) for 2006 to 2008 were obtained from the Oregon Climate Service, Oregon State University, Corvallis, Oregon, USA. Precipitation and temperature data from September prior to sampling periods to the end of each sampling period (i.e. September 2006 – August 2007 and September 2007 – August 2008) was selected to characterize differences in weather conditions between sampling years. Extended weather data were included due to the potential influence of fall and winter precipitation on the hydrologic condition of the pond.

#### Ringed salamanders

Larval ringed salamanders were collected from Kirby’s Pond in Stone County, Missouri, approximately once a week from 20 October 1994 to 11 May, 1995. Four 1-m^2^ quadrats were produced from PVC tubing and were set in place within the pond. Location of each quadrat within the pond at each sampling period was assigned by using randomly generated numbers to determine the compass headings and distance from the perimeter of the pond; quadrats did not overlap. Specimens were collected by sweeping a large net through the quadrat in parallel rows for the entire area, followed by a second sweep of the quadrat with a smaller net in an ‘S’ formation.

Collections from each quadrat were sorted immediately on shore. During the first eight collection periods, the first 10 salamander larvae were euthanized and preserved by submersion in 70% EtOH; for subsequent collections only the first five salamander larvae sorted were kept and preserved. Larval ringed salamanders were measured with vernier calipers (to 0.1 mm) for SVL, MHW, and gape width (GW; gape width measured at posterior edges of the mouth).

Specimens were delivered to the University of Saskatchewan in August, 2012. Larval ringed salamanders were randomly selected from the overall collection and were dissected to analyze contents of the upper digestive tract to distinguish cannibals from non-cannibals. Ingested conspecifics found in the digestive tract of cannibals were not measured for morphometrics due to physical degradation from digestion and long-term preservation. However, hatchling salamanders with partially formed hind limbs collected from the same collection periods as cannibals were used to approximate the morphometrics and stable isotopic values of individuals representing potential prey for cannibals.

For the purpose of comparative morphology between cannibals and non-cannibals, the methods documented in Nyman et al. [19] were followed wherever possible. Cannibals were strictly categorized as individuals that had ingested conspecifics that were clearly identifiable within their digestive tract. Similarly, non-cannibals were categorized as individuals with conspecifics absent from their stomach contents. Non-cannibal specimens selected for comparison with cannibals were restricted to those collected over the same time period as cannibalistic specimens (weeks 3-16) and having a minimum SVL no smaller that of the smallest cannibal (16.3 mm) [19]. Due to the physical degradation of ingested conspecifics occurring as a result of partial digestion, hatchling salamanders collected from weeks 1–2 were used to provide approximate morphometrics and stable isotope values of consumed conspecifics.

Precipitation and temperature data for the collection area (Galena, Missouri) for the October to May collection periods for both 1983-84 and 1994-95 were obtained from the High Plains Regional Climate Center, Lincoln, Nebraska.

Sub-sampling from original collections of both salamanders species were performed using random number sets generated in Excel (Microsoft Corporation, Santa Rosa, CA, USA).

### Stable isotope analysis

Specimens were freeze dried in a Labconco Corp. Freezone® freeze drier for approximately 24 hr. Freeze-dried whole body tadpoles were pulverized to a fine powder, weighed and packaged at the National Hydrology Research Center (NHRC) of Environment Canada, Saskatoon, SK, Canada. Dry powder samples were packaged in ~0.1 mg portions, using Elemental Microanalysis Ltd. 5 × 3.5 mm tin capsules. Samples were subsequently submitted for δ^13^C or δ^15^N analysis to the Stable Isotope Hydrology and Ecology Research Laboratory at NHRC, and the Stable Isotope Laboratory of the Department of Soil Science, University of Saskatchewan. Values for δ^13^C or δ^15^N were expressed relative to Vienna Peedee Belmnite (VPDB) and air, respectively in parts per thousand (‰) [33].

### Statistical analysis

#### Long-toed salamanders

A principle component analysis was performed on SVL, MHW, HL, and PREHW, and the first two components were retained. These components were then analyzed using two 2-sample independent *t*-tests to identify morphological differences between larval populations between 2007 and 2008.

We used two approaches to validate our findings. First, we classified the cannibal status of the individuals based on head morphology and tested for difference in size and stable isotope signatures. Since collections were conducted over four separate sampling periods (June 2007, August 2007, July 2008, August 2008) analysis of larvae was initially conducted independently for each sampling period. Morphometric data was log transformed where it violated parametric assumptions. Two group K-means cluster analyses were used to initially classify salamanders into two groups based on values of head morphology traits (HL, MHW, and PREHW). Larvae classified into the group with the larger head morphology were considered putative cannibal morphs while the group of larvae exhibiting the smaller head morphology were considered putative typical morphs. Separate one-way multivariate analyses of variance (MANOVA) were used to identify differences in log-transformed values of mass and SVL, and in non-transformed δ^13^C and δ^15^N values between groups of salamander larvae in each collection period.

In the second approach, larvae were re-classified into two groups using K-means cluster analyses based on δ^15^N values. Where salamander larvae were classified in this manner, the groups exhibiting the higher δ^15^N values were labelled the cannibal morphs and the group with lower values were labelled as the putative typical morphs. Differences in head shape were also assessed using a discriminate analysis with Wilk’s lambda distribution analysis of allometric transformations of head morphometrics (HL, MHW, and PREHW). We performed an allometric transformation procedure from Reist [34] as used by Nyman et al. [19] to isolate shape components of head dimensions. The predicted variable (Y) was derived for each head morphometric for each individual from the formula Y = 10 k; where k is the log adjusted value of e, and where e = log Y – B(log X – log X_SVL_); where Y is the original head morphometric, B is the regression coefficient of log Y and log SVL, and X_SVL_ is the grand mean of SVL for all larvae. This transformation adjusts the original measurements to values expected for mean body size [19].

Differences in temperature and precipitation for each collection year were compared using a one-way MANOVA.

#### Ringed salamanders

Larval salamanders were classified as cannibals or non-cannibals based on the presence or absence of conspecifics in their digestive tract, respectively. One-way analyses of variance (ANOVA) tests with post-hoc Tukey HSD pairwise comparisons were used to assess differences in log-transformed values of SVL, mass, MHW, and GW among cannibals, non-cannibals and hatchlings. A one-way MANOVA with post-hoc pairwise comparisons was used to identify differences in δ^13^C and δ^15^N values among cannibal, non-cannibal, and hatchling salamanders. Log-transformed values of head morphometrics (MHW and GW) of larvae were tested between groups using two analyses of covariance (ANCOVA) with the log-transformed values of SVL as the covariate.

Head morphometrics were transformed using the aforementioned allometric transformation. A discriminate analysis with Wilk’s lambda distribution test was used on the transformed head morphometrics to explore the relationship between head morphology and observed cannibalism.

Precipitation and temperature data collected from September of the previous year to June of the sampling year. Temperature and precipitation values were tested using a one-way MANOVA. Pond width was recorded at every collection period in 1994-95; however such information was absent from the 1983-84 collection period described by Nyman et al. [19]. To identify the potential difference in pond condition between sampling years we used a predictive regression of precipitation, temperature, and pond widths from 1994-95 to produce representative widths for the pond in 1983-84.

Analyses in both studies were performed using Systat [35]. Outliers were removed where identified through the statistical software. A conservative testing procedure was adopted independently for each experiment by adjusting significance levels using the Holm-Bonferroni correction [36], to reduce the risk of committing a type one error [37]. All figures were produced using SigmaPlot (Systat Software, San Jose, CA, USA).

### Ethical approval

All work was approved by the University of Saskatchewan Animal Ethics Committee under protocols #20070084 and #20120025.
